# The effects of intensive language therapy in aphasic patients

**Published:** 2017-01-05

**Authors:** Ahmad Reza Khatoonabadi, Shohreh Kaviani, Noureddin Nakhostin-Ansari, Mahsa Saadati, Ehsan Shahverdi

**Affiliations:** 1 School of Rehabilitation, Tehran University of Medical Sciences, Tehran, Iran; 2 Department of Speech Therapy, School of Rehabilitation, Neuromuscular Rehabilitation Research Center, Semnan University of Medical Sciences, Semnan, Iran; 3 Department of Physiotherapy, School of Rehabilitation, Tehran University of Medical Sciences, Tehran, Iran; 4 Neuromusculoskeletal Research Center, Iran University of Medical Sciences, Tehran, Iran; 5 Department of Biostatistics, School of Public Health, National Population Studies and Comprehensive Management Institute, Tehran University of Medical Sciences, Tehran, Iran; 6 Students Research Committee, Baqiyatallah University of Medical Sciences, Tehran, Iran; 7 Blood Transfusion Research Center, High Institute for Research Center and Education in Transfusion Medicine, Tehran, Iran

**Keywords:** Aphasia, Intensive Language Therapy, Mississippi Aphasia Screening Test

Aphasia defined as an acquired communication disorder caused by brain damage and characterized by an impairment of language modalities including speaking, listening, reading, and writing.^1^ There are many treatments to restore language functions. Intensive language therapy is one of the most effective treatment approaches. Studies demonstrated that intensive aphasia therapy delivered over 2-3 months were critical to maximize the aphasia recovery and also they reported that higher-intensity therapy provided over a short period results in a significant change in outcome. One novel method of intensive language treating is constraint-induced aphasia therapy (CIAT). In this protocol, patients with aphasia who receive short-term, intensive speech therapy is forced to communicate verbally, and all compensatory strategies (e.g., gesturing, writing, pointing) are restricted.

Effect of CIAT approach on patients naming skill was published previously.^2^ In this study, we decided to evaluate the impact of intense therapy using the CI paradigm on the expressive and receptive index in patients with chronic aphasia.

One of the participants was a 57-year-old male who suffered a left cerebrovascular injury for 7 years before the current investigation. The other participant was a 45-year-old woman who suffered a left cerebrovascular injury for 5 years before the current investigation. In this study, the Mississippi screening aphasia test was the main outcome measure.

Mississippi Aphasia Screening Test (MAST) examines three subtests: (1) expressive index include; naming; automatic speech, repetition, verbal fluency and writing/spelling to dictation, (2) receptive index include: Yes/no accuracy, object recognition, verbal instructions, reading instructions, and (3) total score consists of the expressive and receptive score. The MAST was administered during two phases: (1) baseline (1 time per week for 3 weeks) and (2) treatment (1 time per week for 4 weeks).

The score mean of the receptive index of the first patient was 24.33 at the baseline evaluation which was increased to 31.75 after the intervention, and for the second patient was 29-34. Furthermore, the score mean of the expressive index of the first participant was 9-20.5 and for second patient 22.33-32. Thus, total score mean made an improvement about 18.92 for the first participant and about 14.67 for the second participant. 

We found that CIAT with its characteristics is useful in improving expressive and receptive skills of chronic aphasia patient. Our finding is in consistent with the report of Kurland, et al. which showed that CIAT has a positive effect even in patients with chronic aphasia. CIAT has been shown that is more effective in improving verbal outcome due to cortical reconstruction and neuroplasticity.^3^^-^^5^

## Notes:

**How to cite this article: **Khatoonabadi AR, Kaviani S, Nakhostin-Ansari N, Saadati M, Shahverdi E. The effects of intensive language therapy in aphasic patients. Iran J Neurol 2017; 16(1): 43-4.
